# Characterizing the Host and Symbiont Proteomes in the Association between the Bobtail Squid, *Euprymna scolopes*, and the Bacterium, *Vibrio fischeri*

**DOI:** 10.1371/journal.pone.0025649

**Published:** 2011-10-05

**Authors:** Tyler R. Schleicher, Spencer V. Nyholm

**Affiliations:** Department of Molecular and Cell Biology, University of Connecticut, Storrs, Connecticut, United States of America; New Mexico State University, United States of America

## Abstract

The beneficial symbiosis between the Hawaiian bobtail squid, *Euprymna scolopes*, and the bioluminescent bacterium, *Vibrio fischeri*, provides a unique opportunity to study host/microbe interactions within a natural microenvironment. Colonization of the squid light organ by *V. fischeri* begins a lifelong association with a regulated daily rhythm. Each morning the host expels an exudate from the light organ consisting of 95% of the symbiont population in addition to host hemocytes and shed epithelial cells. We analyzed the host and symbiont proteomes of adult squid exudate and surrounding light organ epithelial tissue using 1D- and 2D-polyacrylamide gel electrophoresis and multidimensional protein identification technology (MudPIT) in an effort to understand the contribution of both partners to the maintenance of this association. These proteomic analyses putatively identified 1581 unique proteins, 870 proteins originating from the symbiont and 711 from the host. Identified host proteins indicate a role of the innate immune system and reactive oxygen species (ROS) in regulating the symbiosis. Symbiont proteins detected enhance our understanding of the role of quorum sensing, two-component signaling, motility, and detoxification of ROS and reactive nitrogen species (RNS) inside the light organ. This study offers the first proteomic analysis of the symbiotic microenvironment of the adult light organ and provides the identification of proteins important to the regulation of this beneficial association.

## Introduction

**Figure 1:**
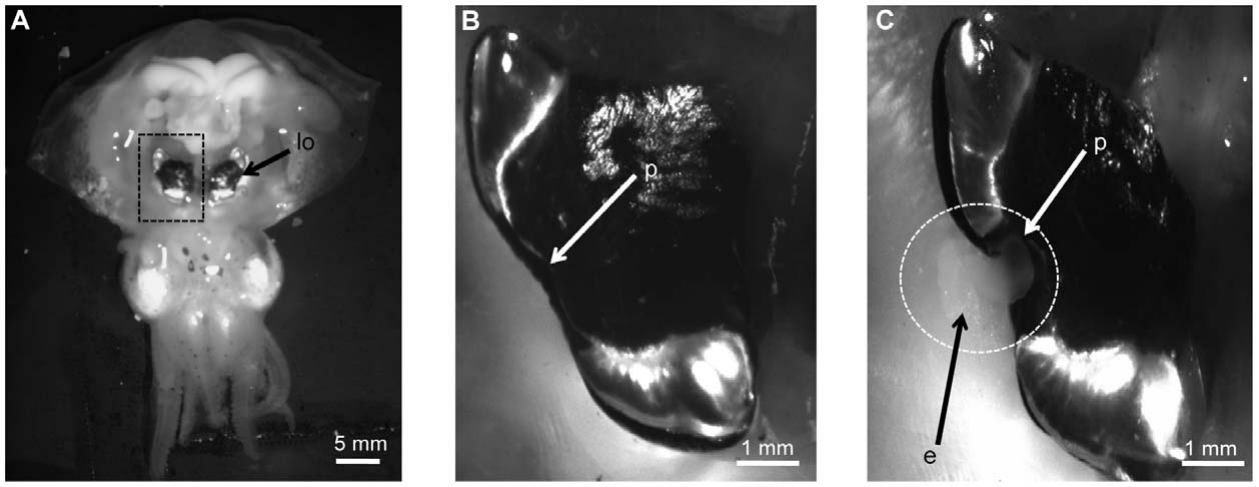
Host and symbiont cells are expelled each morning as a thick exudate. **A.** A ventrally dissected adult squid reveals the bilobed light organ (lo), which is located in the center of the mantle cavity. (Dashed box highlights the region of light organ in **B** and **C**) **B.** One half of the light organ prior to expelling the light organ contents. **C.** One half of the light organ during the venting process. The exudate (e) emerges from a lateral pore (p) as an opaque paste (dotted circle).

The light organ symbiosis between the Hawaiian bobtail squid, *Euprymna scolopes*, and the bioluminescent bacterium, *Vibrio fischeri*, is used as a model association for understanding host/microbe interactions [1]–[3]. Hours after hatching from its egg case, the host is colonized when environmental *V. fischeri* take up residence in epithelia-lined crypt spaces located within a specialized light organ [1]. *V. fischeri* is the sole bacterium that colonizes the light organ and prior research has focused on understanding the mechanisms for establishing and maintaining the high degree of specificity between the partners [1]–[4]. While in the light organ, the bacteria are connected directly to the external environment through ciliated ducts and pores (Fig. 1). This conduit is important as it serves as an interface between the host and the environment and is used in a daily venting of the symbionts. The venting behavior is linked to the nocturnal foraging activities of the host. At night the light organ crypt spaces contain the highest densities of bacteria (10^9^/adult squid; [5]), and the light provided by these symbionts is used to avoid predation [6]. At dawn the host expels 95% of its symbionts from the light organ, while entering a quiescent state in which it buries in the substrate [5], [7]. The remaining bacteria repopulate the crypts ensuring a full complement of symbionts by the following nightfall. This venting mechanism helps regulate the symbiont population in the light organ as well as increases the concentration of *V. fischeri* in the immediate squid habitat, allowing future generations to be colonized [1], [8].

The exudate of adult hosts emerges from the light organ pores as a thick paste-like substance that can be easily collected for experimental analyses (Fig. 1). This material represents the immediate microenvironment of the light organ crypts and is comprised of symbiont cells and a mixed population of host cells (macrophage-like hemoctyes and shed epithelial cells), all surrounded by an acellular matrix [5]. In order to understand the host and symbiont contributions to this microenvironment, previous studies have focused on the cellular and biochemical components of the exudate [5], [9]. Recent work has focused on changes in host and symbiont gene expression during the daily rhythm within the light organ [10]. Transcriptome analyses at different time points during the day/night revealed dynamic changes both metabolically and physiologically for the host and symbiont, and identified a large number of differentially expressed genes [10]. In addition, microscopy at these time points revealed that the crypt epithelium also undergoes morphological changes whereby apical surfaces are blebbed into the crypt spaces [10]. Many of these gene expression and cellular changes were most dramatic in the hours just before and after dawn, reflecting the dynamic turnover that occurs in the light organ upon venting.

In this study, we employed a number of techniques to characterize the host and symbiont proteomes of the adult light organ microenvironment at dawn when the association undergoes a dramatic reduction in symbiont population. To date, proteomic analyses of the squid/*Vibrio* association are limited. A previous study used two-dimensional polyacrylamide gel electrophoresis (2D-PAGE) to reveal numerous differences in the soluble proteins present in the light organs of juvenile aposymbiotic (uncolonized) and symbiotic (colonized) squid during the development of the symbiosis, however no proteins were identified [11]. Recent advances in proteomics, including multidimensional protein identification technology (MudPIT), have provided the tools to allow the identification of a large number of host and symbiont proteins in the squid/*Vibrio* association for the first time [12], [13]. MudPIT utilizes strong cation exchange chromatography (SCX) to separate peptides by charge prior to liquid chromatography tandem mass spectrometry (LC MS/MS), thus increasing the number of identified peptides. In this study we utilized MudPIT, in addition to 1D- and 2D-PAGE, to describe both the host and symbiont proteomes in the light organ exudate and the surrounding host epithelial tissue. These analyses identified components of the host's innate immune system as well as numerous proteins involved in the detoxification of reactive oxygen species (ROS). Symbiont proteins detected were involved in stress responses, quorum sensing, motility, and two-component signaling pathways. Our data also highlight many proteins that are presently uncharacterized with regard to the squid/*Vibrio* symbiosis. Identifying the host and symbiont proteins present in the light organ represents a first step to understanding key functional aspects of the association's molecular dialogue that is responsible for maintaining this highly specific relationship and complements a number of other molecular and genetic techniques that have been applied to this symbiosis.

## Materials and Methods

### Ethics statement

*Euprymna scolopes* is an invertebrate and is not regulated by animal care regulations in the United States. All field collection of research animals was done in accordance with state and federal regulations. The State of Hawaii does not require collection permits for this species outside of marine reserves. None of the animals collected for this study were caught/collected within a marine reserve or regulated area.

### General methods

Adult animals were collected in shallow sand flats of Maunalua Bay, Oahu, HI by dip net and were either maintained in the laboratory in re-circulating natural seawater aquaria at the Hawaii Institute of Marine Biology or at the University of Connecticut with artificial seawater (ASW, Instant ocean) at 23°C. All animals were acclimated at least 48 hours under laboratory conditions and kept on an approximate 12 hr light/12 hr dark cycle before sample collection. *V. fischeri* strain ES114 was grown in saltwater tryptone (SWT) at 28°C as previously described [14].

### Exudate and central core collection

Exudate was collected as previously described [5]. Briefly, adult squid were anesthetized in a 2% ethanol/seawater solution and ventrally dissected under red light within minutes prior to dawn. A light stimulus (150 W halogen light) was used to induce venting behavior. Within 1 h, the squid had expelled the light organ contents, which were collected with a 10-µl disposable micropipette (Drummond Scientific Company) and stored on ice after the addition of a 1× protease inhibitor cocktail according to the manufacturer's protocol (Sigma Aldrich, P2714). Post-vented central cores were also dissected and removed from the light organ. All samples were flash frozen with liquid nitrogen and stored at −80°C until further analysis. No differences were detected between samples collected from animals maintained at either the Hawaii Institute of Marine Biology or at the University of Connecticut (data not shown).

### Gel-based proteomic methods

#### Exudate sample preparation for 1D- and 2D-polyacrylamide gel electrophoresis

For PAGE applications, symbiont cells from freshly collected light organ exudate were separated from the soluble fraction, a source of host proteins, by centrifugation (Eppendorf 5810 R, 5,000 rpm, 10 minutes, 4°C). The symbiont pellet was washed three times with 0.22 µm filtered ASW to remove additional soluble proteins. Symbiont proteins were extracted by a modified method from Ho and Hsu [15]. Briefly, 10 consecutive liquid nitrogen freeze/thaw cycles were performed in the presence of a 1× protease inhibitor cocktail (Sigma Aldrich, P2714) with 80 mM Tris, pH 8.0 for cell lysis. After separation from the bacterial pellet, soluble host proteins were quantified (see below) and stored until further analysis. For cultured *V. fischeri*, cells were grown to early stationary phase [14] and proteins were extracted as described for the symbiont exudate pellet. Protein concentrations of separate symbiont exudate and host soluble fractions, as well as culture-grown *V. fischeri*, were determined spectrophotometrically using the method of Whitaker and Granum [16] and/or a Bradford assay (Bio-Rad). Typically, protein extractions of exudate resulted in 10–20 µg of protein combined from the host soluble and symbiont pellet fractions. Comparison of 2D-PAGE gels from soluble proteins of culture-grown *V. fischeri* and the host soluble fraction of the exudate demonstrated that the soluble host fraction was devoid of bacterial proteins (data not shown).

#### 1D-polyacrylamide gel electrophoresis of light organ exudate

Between 10 and 20 µg of exudate protein from either the host (soluble protein separated from bacterial pellet) or symbiont fraction (bacterial pellet) were resolved with 12.5% polyacrylamide gels (Bio-Rad). Electrophoresis was performed with a Hoeffer 250 mini-gel apparatus at 23 mA or a Bio-Rad Mini PROTEAN® Tetra cell at 200 V for 30 minutes. Gels were either stained with Bio-Rad Brilliant Blue Coomassie R-250 or a Bio-Rad Silver Stain Plus Kit (Bio-Rad). 1D-PAGE of both the soluble host fractions and the bacterial pellets were shown to be reproducible (n = 3, separate and fractionated pooled exudate samples for each; data not shown).

#### 2D-polyacrylamide gel electrophoresis of light organ exudate

2-D PAGE was performed using the Amersham Pharmacia Biotech Multiphor II system as previously described [11]. 40 µg of pooled exudate protein from either the soluble host fraction or the bacterial pellet, originating from 2 or more adult squid or culture-grown *V. fischeri* cells, were denatured 1∶4 in 9 M urea, 1% DTT, 2% Pharmalyte 3–10, 0.5% Triton-X-100, 0.14% phenylmethylsulfonyl fluoride, loaded onto a first dimension gel strip with an immobilized pH gradient (4–7) and focused over a 20 hour period. Samples were then separated by molecular weight on pre-cast 12% to 14% polyacrylamide gradient gels (GE Healthcare Life Sciences). Gels were silver stained as previously described [11], [17]. 2D-PAGE from the soluble host fractions and the bacterial pellets or culture-grown cells were deemed to be highly reproducible (n = 3, separate and fractionated pooled exudate samples; data not shown). For comparison, 2D gels were visually aligned and similarities and differences of the molecular weights and individual protein species were noted. Five spots of interest from the 2D-PAGE gel of the exudate bacterial pellet were excised and successively washed in 50% acetonitrile, 50% acetonitrile/50 mM NH_4_HCO_3_, and 50% acetonitrile/10 mM NH_4_HCO_3_. The five gel spots were then dried by speed vacuum (Eppendorf Concentrator 5301) and resuspended in 10 mM NH_4_HCO_3_. Digestion was completed with 0.1 µg trypsin (Promega, V5111) per each 15 mm^3^ of gel in a final volume of 35 µl of 10 mM NH_4_HCO_3_ at 37°C for 24 hours. The digested samples were stored at −80°C until submission to the W. M. Keck Biotechnology Resource Laboratory, Yale University, for LC MS/MS (See below, Mass spectrometry proteomics).

### Mass spectrometry proteomic methods

#### Protein preparation for multidimensional protein identification technology and liquid chromatography tandem mass spectrometry

For MudPIT and LC MS/MS, pooled host and symbiont fractions from freshly collected light organ exudate were combined and quantified as described above. Additionally, central cores were homogenized in the presence of a 1× protease inhibitor cocktail (Sigma Aldrich, P2714) with 80 mM Tris, pH 8.0 using a ground-glass homogenizer. Proteins from central cores were collected from the supernatant of the homogenate after centrifugation (Eppendorf 5810 R, 14,000 rpm, 30 minutes, 4°C) and quantified as described above. Extractions of the central core tissue resulted in approximately 20 µg of soluble protein per central core. Total protein from pooled exudate samples (50 µg, n = 7 squid and 100 µg, n = 7 squid) and pooled central core samples (40 µg, n = 3 squid) were precipitated with 10% trichloroacetic acid (Fisher Scientific) at 4°C overnight. The protein precipitates of the exudates and central cores were collected by centrifugation (Eppendorf 5810 R, 11,000× *g*, 30 minutes, 4°C) and washed twice with ice-cold acetone. The protein pellets were briefly air-dried and then solubilized in 25 µl of 8 M urea, 0.4 M ammonium bicarbonate, pH 8.0. Both samples were reduced and alkylated with 5 µl of 45 mM dithiothreitol (DTT; Acros Organics) at 37°C for 20 minutes and 5 µl of 100 mM iodoacetamide (Acros Organics) at room temperature in the dark for 20 additional minutes. Sequencing grade trypsin was added 1∶15 (w/w enzyme to protein; Promega, V5111). The solutions were diluted in water to 100 µl (2 M urea final concentration). Both samples were digested at 37°C for 18–24 hours and then stored at −80°C until submission to the W. M. Keck Biotechnology Resource Laboratory, Yale University for LC MS/MS.

For MudPIT, tryptic digests of pooled exudate proteins from *E. scolope*s underwent strong cation exchange (SCX) on an Applied Biosystems Vision Workstation at the W. M. Keck Biotechnology Resource Laboratory at Yale University. During SCX, peptides were separated by charge into fractions, which were then analyzed by LC MS/MS. MudPIT analyses of separate pooled exudate samples were run in duplicate. The first analysis used 10 SCX fractions (50 µg, n = 7 squid) and the second used 20 SCX fractions (100 µg, n = 7 squid), allowing greater coverage of lower abundance peptides. The central core sample (40 µg, n = 3 squid) and symbiont exudate 2D-PAGE spots (n = 5 spots) were analyzed by one-dimensional LC MS/MS (see below).

For SCX, the tryptic digests of pooled exudate proteins were acidified with 2 µl of 1 M phosphoric acid. A 2.1 mm×200 mm PolySULFOETHYL A™ column (PolyLC Inc.) was used to establish a linear gradient for 118 minutes. The gradient was maintained in 10 mM potassium phosphate, 25% acetonitrile (pH 3.0) and the same buffer with the addition of 1 M potassium chloride. Fractions were collected every 2 minutes at a flow rate of 150 µl/min. All fractions were dried, dissolved in 5 µl of 70% formic acid, and diluted to 15 µl in 0.1% trifluoroacetic acid for subsequent LC MS/MS.

#### Liquid chromatography tandem mass spectrometry

LC MS/MS of each exudate SCX fraction, central core peptides, and 2D gel spot peptides was completed at the W. M. Keck Biotechnology Resource Laboratory at Yale University. A LTQ Orbitrap mass spectrometer (Thermo Fisher Scientific) equipped with a Waters nanoAcquity UPLC system operated with a Waters Symmetry® C18 180 µm×20 mm trap column, and a 1.7 µm, 75 µm×250 mm nanoAcquity™ UPLC™ column (35°C) was used for peptide separation. Trapping was performed at 15 µl/min with Buffer A (100% water, 0.1% formic acid) for 1 minute. Peptide separation was performed at 300 nl/min with Buffer A and Buffer B (100% CH_3_CN, 0.075% formic acid); a 51 minute linear gradient was established starting with 5% Buffer B, increasing to 50% B at 50 minutes, and finally 85% B at 51 minutes. MS was acquired in the Orbitrap using 1 microscan followed by four data dependent MS/MS acquisitions. Neutral loss scans (MS^3^) were also obtained for 98.0, 49.0, and 32.7 amu.

#### Data analysis

All MS/MS spectra were analyzed using the Mascot algorithm for uninterpreted MS/MS spectra [18]. The Mascot Distiller program used the MS/MS spectra to generate Mascot compatible files by combining sequential MS/MS scans from profile data that have the same precursor ion. A charge state of +2 and +3 were preferentially located with a signal to noise ratio of 1.2 or greater and a peak list was created for database searching. The peak list was searched by Mascot using *V. fischeri* amino acid sequence and juvenile *E. scolopes* light organ expressed sequence tag (EST) databases [19]. Search parameters included partial methionine oxidation, carboxamidomethylated cysteine, a peptide tolerance of ±20 ppm, MS/MS fragment tolerance of ±0.6 Daltons (Da), and peptide charges of +2 or +3. Normal and decoy database were also searched. Mascot significance scores are based on a MOlecular Weight SEarch (MOWSE) scores and rely on multiple matches of more than one peptide to the same protein [20]. The MOWSE based ions score is equal to (−10)*(Log_10_P), where P is the absolute probability that a match is random. For a match to be significant, the probability of it being a random match should be below 5% (E-value<0.05) [21]. The protein threshold score depends on the size of the database being searched, therefore, Mascot determined that scores greater than 68 were significant when searching the juvenile light organ EST database and scores greater than 48 were significant when searching the *V. fischeri* ES114 amino acid database. Proteins were considered identified when 2 or more peptides matched the same protein and if the Mascot score was above the respective significance threshold. Proteins with putative identifications contained two or more peptide matches, but had a Mascot score below the threshold for the respective database (E-value>0.05).

Mascot also calculates the exponentially modified protein abundance index (empai) which estimates the abundance of protein species by using the number of peptides detected in the analysis compared to the number of possible peptides for a particular protein [22], [23].

Host proteins identified by Mascot using the juvenile light organ EST database were further analyzed using the Bioinformatics Utility for Data Analysis of Proteomics using ESTs (BUDAPEST) which removed any peptides matching to non-coding reading frames [24]. BLASTx (E-value cutoff 10^−6^) against the NCBI nr database was used to determine the top protein hit for each EST [25]. In addition, BUDAPEST calculated a peptide score for each protein identified. This score was equal to the number of correct reading frame peptides squared divided by the total number of peptides (all reading frames) identified for that EST. BUDAPEST scores greater than 1 can be considered significant, however, in our study scores of 2 or greater were chosen to represent significant protein identifications.

## Results

**Figure 2:**
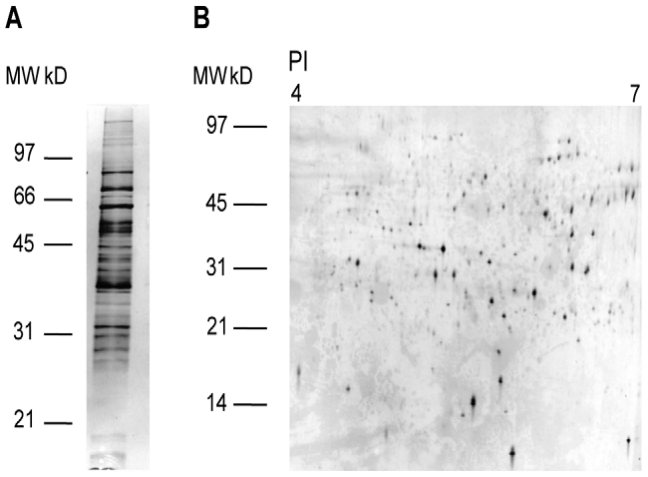
PAGE analysis of the soluble host fraction from light organ exudate. **A.** 1D-PAGE of the host fraction of the exudate on a 12.5% polyacrylamide gel. **B.** 2D-PAGE of the host fraction of the exudate on a 12–14% polyacrylamide gel.

**Figure 3:**
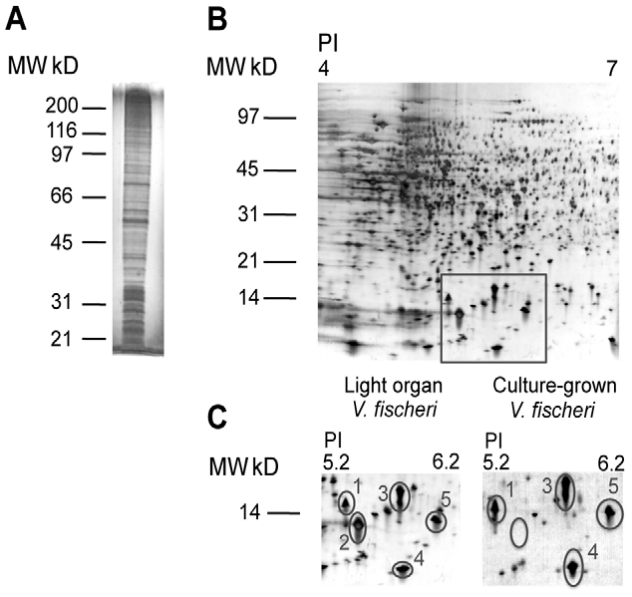
PAGE analysis of the soluble proteins originating from the symbiont fraction of the exudate. **A.** 1D-PAGE of symbiont fraction of the exudate on a 12.5% polyacrylamide gel. **B.** 2D-PAGE of the symbiont fraction of the exudate on a 12–14% polyacrylamide gel. Black box highlights the region of the gel compared in C. **C.** 2D-PAGE comparison of bacterial soluble proteins from the exudate and culture-grown *V. fischeri*. Numbered protein spots were identified by LC MS/MS (Table 1).

Exudate samples collected from adult *E. scolopes* light organs were analyzed using a number of proteomic techniques. 1D- and 2D-PAGE revealed that the host soluble fraction of the exudate, derived from host hemocytes and apical surfaces of shed light organ crypt epithelial cells, was comprised of a complex mixture of proteins and peptides, the majority of which are represented between the isoelectric points of 4 to 7 and a size of 7 to 100 kilodaltons (kD) (Fig. 2A, B). Similar analyses of the symbiont fraction of the exudate also revealed a complex protein profile (Fig. 3A, B). Comparison of the host and symbiont PAGE gels support previous observations that the exudate appears enriched in bacteria. When comparing proteins expressed by *V. fischeri* in the light organ to proteins expressed by *V. fischeri* in culture, a protein with an isoelectric point of 5.5 and a molecular weight of 10 kD was present in the light organ, but not in solubilized proteins from culture-grown *V. fischeri* (Fig. 3C). The protein spot of interest (Fig. 3C, spot 2) and four surrounding protein spots (common to both the light organ and culture) were excised and identified by LC MS/MS (Table 1). The unique symbiont light organ protein was determined to be a quorum sensing-regulated protein (QsrP), which has been previously identified as being expressed by *V. fischeri* in the light organ, but remains functionally uncharacterized [26].

**Table 1:** Exudate proteins identified by LC MS/MS from symbiont 2D-PAGE analysis (Fig. 3).

Spot	Mw (kD)/pI^a^	Top Protein Hit to NCBI nr Database	ORF^b^	gi	Score^c^
1	13.3/5.4	50S ribosomal protein L9	VF_2310	59712917	1045
2	11.0/5.5	Quorum sensing regulated protein QsrP	VF_A1058	59714241	628
3	14.7/5.7	Transcriptional dual regulator H-NS	VF_1631	59712238	712
4	8.8/5.7	Cold shock protein	VF_2561	59713168	473
5	9.3/6.1	30S ribosomal protein S6	VF_2312	59712919	501

a Predicted molecular weight (Mw) and isoelectric point (pI) for the proteins identified.

b Open reading frame (ORF) locations of the respective genes on the chromosomes of *V. fischeri*.

c Scores were assigned by Mascot. Scores greater than 83 were significant (E-value<0.05) for searches of the NCBI nr database.

In an effort to further characterize the proteins expressed by the host and symbiont we utilized shotgun proteomic techniques (LC MS/MS and MudPIT). These methods allowed us to putatively identify a combined 1581 host and symbiont proteins present in the light organ. For MudPIT, light organ exudate samples of 10 or 20 SCX peptide fractions (see Materials and Methods) were analyzed (Table 2). In addition, to increase our representation of host proteins we analyzed post-vented central cores by single fraction LC MS/MS (Table 2). A total of 870 unique symbiont proteins were putatively identified by Mascot from all the light organ samples (exudates and central cores; Table S1). 516 of these proteins were above the significance threshold set by Mascot (E-value<0.05). For the host, we utilized BUDAPEST, a software program developed specifically to identify proteins in the correct open reading frame in cases when only EST databases are available [24]. 676 host proteins with more than 2 peptides matching to the correct reading frame and a BUDAPEST score of greater than or equal to 2 were identified from combining the LC MS/MS and MudPIT data of the exudate and central core samples (Table S2).

**Table 2:** Number of host and symbiont proteins identified by shotgun proteomics^a^.

Sample	*Vibrio fischeri*	*Euprymna scolopes* b
Exudate (10 Fractions)	214	163
Exudate (20 Fractions)	708	591
Central Core (1 Fraction)	90	234
Total unique proteins	870	711

a Protein identifications required 2 or more peptides per protein. Total unique proteins summarize the combined unique proteins from all samples. Numbers include putative identifications (see Materials and Methods).

b Host protein identifications counts are post-BUDAPEST analysis.

All host and symbiont proteins, including putative identifications, were organized functionally according to the Clusters of Orthologus Groups database (COG and KOG; Fig. S1, Table S1, Table S2) [27], [28]. In order to achieve a more thorough understanding of the functions represented by the proteins in our data, we first analyzed the relative abundance of each symbiont protein. The 25 most abundant symbiont proteins determined by empai include the protein subunits of luciferase (LuxAB), QsrP, alkyl hydroperoxide reductase C22 (AhpC), and several cold shock proteins (Table 3). Our analyses also identified a number of symbiont proteins related to functions involved in stress responses, quorum sensing, motility, and signaling pathways, all of which have been previously implicated as being important in the squid/*Vibrio* association (Table 4, Table S3; see discussion). Several of these identified proteins including AhpC and the cold shock proteins have symbiotic roles yet to be characterized.

**Table 3:** The 25 most abundant symbiont proteins present in light organ exudates and central cores identified by MudPIT and LC MS/MS in descending empai order (excluding ribosomal proteins).

#	gi	ORF^a^	Gene	Protein Name	empai	Score^b^
1	59714104	VF_A0921	luxA	luciferase alpha chain LuxA	28.74	2840
2	59712269	VF_1662	-	DNA-binding protein	21.59	257
3	59712346	VF_1739	acpP	acyl carrier protein	19.95	290
4	59714241	VF_A1058	qsrP	LuxR-regulated periplasmic protein QsrP	13.61	691
5	59712582	VF_1975	ahpC	alkyl hydroperoxide reductase, C22	10.54	994
6	59711657	VF_1050	-	hypothetical protein	9.35	179
7	59711351	VF_0744	ybeD	hypothetical protein	7.94	194
8	59712374	VF_1767	cspD	DNA replication inhibitor	7.66	309
9	59714005	VF_A0822	-	hypothetical protein	7.55	101
10	59711823	VF_1216	infC	protein chain initiation factor IF-3	6.86	378
11	59711844	VF_1237	ihfA	integration host factor subunit alpha	6.41	193
12	59712727	VF_2120	arcA	two-component response regulator	6.32	799
13	59711232	VF_0625	Ndk	nucleoside diphosphate kinase	5.92	245
14	59712758	VF_2151	-	iron(III) ABC transporter	5.3	1109
15	59711304	VF_0697	-	putative lipoprotein	5.22	72
16	59711049	VF_0442	Pgk	phosphoglycerate kinase	5.08	1747
17	59711497	VF_0890	grxA	glutaredoxin 1	4.88	216
18	59710869	VF_0262	rpoA	DNA-directed RNA polymerase alpha	4.84	647
19	59713778	VF_A0595	-	cold shock protein	4.78	273
20	59712703	VF_2096	-	hypothetical protein	4.77	146
21	59714277	VF_A1094	cspG	DNA-binding transcriptional regulator	4.64	776
22	59712568	VF_1961	Tsf	elongation factor Ts	4.36	794
23	59714103	VF_A0920	luxB	luciferase beta chain LuxB	3.93	1434
24	59710881	VF_0274	-	immunogenic protein	3.87	442
25	59711114	VF_0507	deoD	purine nucleoside phosphorylase	3.86	140

a Open reading frame (ORF) locations of the respective genes on the chromosomes of *V. fischeri*.

b Scores were assigned by Mascot. Scores greater than 48 were significant for *V. fischeri*.

**Table 4:** Symbiont proteins detected in light organ exudates and central cores by MudPIT and LC MS/MS^a^.

Category	gi	ORF^b^	Protein Name	Score^c^
*Quorum Sensing*				
	59714104	VF_A0921	luciferase alpha chain LuxA	2840
	59714103	VF_A0920	luciferase beta chain LuxB	1434
	59714241	VF_A1058	LuxR-regulated periplasmic protein QsrP	691
	59711956	VF_1349	subtilisin-like serine protease	491
	59714105	VF_A0922	acyl transferase LuxD	463
	59714077	VF_A0894	putative surface protein	304
	59714106	VF_A0923	acyl-CoA reductase LuxC	225
	59711152	VF_0545	S-ribosylhomocysteinase LuxS	167
	59712332	VF_1725	secretory tripeptidyl aminopeptidase	123
	59712784	VF_2177	LitR	78
	59714102	VF_A0919	long-chain-fatty-acid ligase LuxE	62
	59712585	VF_1978	AcfA-like protein	50
	59711772	VF_1165	macrolide ABC transporter	45
	59714108	VF_A0925	LuxR	21
*Oxidative Stress*				
	59712582	VF_1975	alkyl hydroperoxide reductase, C22	994
	59711509	VF_0902	thioredoxin reductase	238
	59713192	VF_A0009	hydroperoxidase HPII(III) KatA	184
	59711528	VF_0921	superoxide dismutase, Fe	83
	59712527	VF_1920	thioredoxin-dependent thiol peroxidase	54
	59712923	VF_2316	nitric oxide dioxygenase	46
	59714073	VF_A0890	thioredoxin peroxidase	19
*Two-Component Signaling*				
	59712727	VF_2120	ArcA	799
	59713744	VF_A0561	two component response regulator	207
	59712949	VF_2342	periplasmic protein CpxP	154
	59712177	VF_1570	TorR	111
	59711061	VF_0454	transcriptional regulator VpsR	83
	59712516	VF_1909	DNA-binding response regulator NarP	63
	59712234	VF_1627	response regulator GacA	59
	59710721	VF_0114	osmolarity response regulator OmpR	54
	59712981	VF_2374	two-component response regulator	46
	59713399	VF_A0216	two component response regulator	36
	59714199	VF_A1016	two component sensory histidine kinase	32
	59711755	VF_1148	response-regulatory protein YehT	23
	59712226	VF_1619	hybrid sensory histidine kinase TorS	20
	59712008	VF_1401	sigma-54 dependent response regulator	16
	59712950	VF_2343	DNA-binding response regulator CpxR	15
*Flagellar-related proteins*				
	59712463	VF_1856	FlrA	107
	59712488	VF_1881	flagellar anti-sigma-28 factor FlgM	81
	59712471	VF_1864	flagellin	65
	59712473	VF_1866	flagellin	62
	59712478	VF_1871	flagellar basal body L-ring protein	34
	59711322	VF_0715	flagellar motor protein MotB	29
	59712477	VF_1870	flagellar basal body P-ring protein	18
	59712484	VF_1877	flagellar basal body rod protein FlgB	14

a For more complete information on MudPIT and LC MS/MS symbiont protein identifications refer to Table S1 and Table S3.

b Open reading frame (ORF) locations of the respective genes on the chromosomes of *V. fischeri*.

c Scores were assigned by Mascot. Scores greater than 48 were significant for *V. fischeri*.

Host proteins detected in the light organ highlight the innate immune system, oxidative stress, and signaling pathways (Table 5). Identified proteins include those involved with the NFκB signaling pathway and the recognition of microbial associated molecular patterns (MAMPs) such as peptidoglycan recognition proteins (PGRPs) and a carbohydrate binding protein, galectin (Table 5). Proteins related to oxidative stress consist of superoxide dismutase, peroxiredoxins and numerous peroxidases, including the *E. scolopes* halide peroxidase (EsHPO) (Table 5; see discussion). Additionally, several host proteins involved iron-sequestration were detected in the light organ.

**Table 5:** Host proteins detected in light organ exudates and central cores by MudPIT and LC MS/MS^a^.

Category	gi	Top protein hit to NCBI nr database	e-value^b^	Score^c^
*Immunity*				
	225906399	Galectin [*Pinctada fucata*]	4E-69	10
	63033995	Peptidoglycan recognition protein 2 [*E. scolopes*]	1E-121	7
	223670954	C3 precursor [*Nematosella vectensis*]^d^	4E-12	6
	144952812	Thioester-containing protein [*Chlamys farreri*]	4E-14	6
	113931358	NFKB repressing factor [*Xenopus tropicalis*]	2E-17	4
	42741753	Importin alpha 3 [*Aplysia californica*]	9E-6	4
	63033997	Peptidoglycan recognition protein 3 [*E. scolopes*]	9E-49	3
	85822201	TEP2 [*Glossina morsitans morsitans*]	3E-16	2
*Oxidative stress*				
	306451460	Thioredoxin peroxidase [*Cristaria plicata*]	1E-92	13
	110734438	Superoxide dismutase [*Haliotis discus discus*]	7E-61	7
	229366436	Peroxiredoxin-5 [*Anoplopoma fimbria*]	2E-51	5
	67083759	Glutathione-type peroxidase [*Ixodes scapularis*]	8E-50	5
	2239176	Melanogenic peroxidase [*Sepia officinalis*]	3E-59	4.5
	209171295	Peroxiredoxin 4 precursor [*Biomphalaria glabrata*]	1E-101	4
	159008	Halide peroxidase [*Euprymna scolopes*]	1E-141	4
	157136354	Peroxiredoxins, prx-1, prx-2, prx-3 [*Aedes aegypti*]	5E-72	3.2
	77166828	Glutathione peroxidase [*Rhipicephalus microplus*]	4E-62	3
	149688674	Peroxiredoxin [*Chlamys farreri*]	2E-58	2
	126697356	Thioredoxin peroxidase 2 [*Haliotis discus discus*]	1E-54	2
*Iron-Binding*				
	318067980	Transferrin [*Ictalurus punctatus*]	4E-34	6
	4768842	Ferritin [*Enteroctopus dofleini*]	7E-74	3
	157786780	Melanotransferrin [*Rattus norvegicus*]	6E-21	3

a For more complete information on MudPIT and LC MS/MS host protein identifications refer to Table S2.

b E-value represents the alignment of the light organ EST with the top protein hit in the NCBI nr database.

c Scores were assigned by BUDAPEST and correlate the number of reading frame peptides matched to the light organ EST to the number of overall peptides. Scores greater than 2 were significant.

d No alignment with *Euprymna scolopes* C3 (Putatively identified as a thioester-containing protein; see discussion).

## Discussion

The daily expulsion of *V. fischeri* from the light organ of *E. scolopes* provides a unique opportunity to characterize the interactions between the host and symbiont in a natural microenvironment. Previous analyses of this exudate have focused on the cellular and biochemical composition of the expelled matrix [5], [9]. In this study we characterized the light organ exudate and surrounding epithelial proteome using MudPIT and PAGE. A total of 1581 unique host and symbiont proteins were putatively identified from the light organ, offering the first proteomic analyses of this symbiotic microenvironment.

### Innate immune system

MAMPs and host pattern recognition receptors (PRRs) are two components underlying host/microbe interactions and are significantly involved in the development of this association [29]. MAMPs including lipopolysaccharide (LPS), and peptidoglycan and its derivatives, function in determining the specificity of the squid/*Vibrio* symbiosis as well as initiating morphogenetic changes to the light organ [30]–[32]. We identified several host proteins related to pattern recognition in both the exudate and central core tissues (Table 5). *E. scolopes* PGRP2 and 3 (EsPGRP2 and EsPGRP3) are involved in detecting peptidoglycan, a major cell wall component of bacteria [33]. EsPGRP2 is secreted into the crypts of the light organ where it is thought to degrade tracheal cytotoxin (TCT), a monomer of peptidoglycan [34]. The role of EsPGRP3 in the symbiosis is currently under investigation, but has been detected in adult and juvenile hemocytes (unpublished data). Certain carbohydrates, such as beta-galactosides, are another type of MAMP that are recognized by carbohydrate binding proteins known as galectins [35]. A putative galectin was identified in both the exudate and central core tissue (Table 5, Table S2) and may have an uncharacterized role in the squid/*Vibrio* symbiosis.

Aside from PRRs and MAMPs, cellular adhesion is often important for host/microbe cell-to-cell interactions. Outer membrane proteins (OMPs) are localized at the bacterial cell surface and are good candidates for mediating recognition between the partners. OmpU, a symbiont outer membrane protein that we have identified in the light organ (Table S3), was shown to be important in mediating adhesion to adult host hemocytes and during the early stages of colonization [36], [37]. Other OMPs identified, such as a hypothetical protein VF_1010, have roles yet to be characterized in binding and adhesion, but may have similar functions (Table S3). Understanding how the symbiont outer membrane proteome varies in the light organ vs. the free-living environment and between symbiosis-competent and incompetent strains may shed light on mechanisms of mediating specificity in this symbiosis.

An immune pathway highlighted by our proteomic data includes NFκB signaling (Table 5). The role of NFκB signaling during the establishment of the squid*/Vibrio* symbiosis is currently under investigation, however, many important members of the pathway have been identified from juvenile light organ ESTs [33]. We detected NFκB repressing factor (see below) and importin alpha 3, a protein involved in shuttling proteins into the nucleus by recognizing nuclear localization signals (Table 5) [38]. *In vitro* and *in vivo* studies using cancer cell lines reveal this protein is a member of the NFκB signaling pathway and aids in the transport of NFκB transcription factors into the nucleus [39].

Recently, *E. scolopes* has been shown to have a complement pathway that in other systems is involved with mediating inflammation and opsonization [29], [40], [41]. The function of this pathway has yet to be described in the squid/*Vibrio* symbiosis, however, we detected putative components of the complement cascade in both the exudate and the central core (Table 5, Table S2). Although one of these identifications was annotated as a complement component C3 precursor (Table 5, Table S2), closest to the cnidarian *Nematostella*, further analysis of these peptides using *E. scolopes* transcriptomic data revealed that this protein did not align with the previously described *E. scolopes* C3 (data not shown). Instead, this protein, along with two others, were identified as thioester-containing proteins (TEPs). Among invertebrates, TEPs play an important role in innate immune response as members of the complement system or as protease inhibitors [42], [43].

### Reactive oxygen and nitrogen stress response

The chemical microenvironment of the light organ crypts likely influences the maintenance of the association and helps to ensure specificity. Although oxygen is critical for the bioluminescence reaction, reactive oxygen species (ROS) and toxic oxygen intermediates have been shown to be abundant in the light organ [44]. Host-derived ROS, such as hypohalous acid, are thought to play key roles in initiation and persistence of the squid/*Vibrio* symbiosis [44]. Hypohalous acid, produced by an abundant light organ peroxidase similar to a halide peroxidase, is believed to help to create an oxidative environment that *V. fischeri* must overcome to colonize the host [45], [46]. In addition to the previously described EsHPO, a number of other host peroxidases were present, suggesting that additional ROS may be important to this association (Table 5). Peroxiredoxins are antioxidant proteins, which are abundant in the host proteome and have been shown to detoxify reactive molecular species derived from oxygen and nitrogen [47], [48]. Therefore, these ROS mediators may indicate a means by which the host protects its own tissues in the oxidative microenvironment of the light organ.

Another role of host ROS may be maintaining specificity by preventing non-symbiotic bacteria and potential pathogens from infecting the host. The light organ crypts are open to the environment via pores on the surface of the light organ, yet *V. fischeri* is thought to be the sole symbiont of this highly specific association [1]. Proteins expressed by the symbiont reveal functions involved with protecting cells from host ROS (Table 4). *V. fischeri* utilizes a periplasmic catalase (*katA*) to sequester hydrogen peroxide from the host, which can be used by EsHPO to generate hypohalous acid [49]. We identified, in addition to KatA, the antioxidant enzymes AhpC and thioredoxin-dependent thiol peroxidase (Bcp) (Table 4). A *V. fischeri katA* mutant showed no additional catalase activity in culture suggesting that KatA is the major scavenger of H_2_O_2_ [49]. The additional antioxidant proteins identified in this study may indicate a mechanism by which the symbiont can protect itself from other types of ROS or RNS. AhpC, a peroxiredoxin capable of reducing hydrogen peroxide, organic peroxides, and peroxynitrite, is the most abundant antioxidant symbiont protein present in the light organ (Table 3). In *Vibrio vulnificus*, AhpC functions along with another subunit, AhpF, which supplies the reducing equivalents in the form of NADH, to reduce peroxides [50]. However, AhpF is absent from the *V. fischeri* genome, suggesting that another protein is necessary to reduce peroxides by this pathway. Studies involving *Treponema pallidum* show that thioredoxin reductase can substitute for organisms lacking an AhpF homolog [51]. For *V. fischeri*, a thioredoxin reductase FAD/NAD(P)-binding protein (TrxB) was present in our MudPIT data (Table 4) and may have the potential of interacting with AhpC. Along with AhpC, proteins implicating that *V. fischeri* also detoxifies RNS, include nitric oxide dioxygenase (Hmp), and two peptide-methionine (S)-S-oxide reductases (MsrA and VF_A0005; Table S1) [52], [53].

Reactive nitrogen species, such as nitric oxide (NO), contribute to signaling and development in the squid/*Vibrio* symbiosis [54]. The role of NO as a toxic product to pathogens has been well studied; however, the function of NO in beneficial associations has only been recently analyzed [54], [55]. In juvenile squid the epithelial tissue lining the ducts entering the light organ crypts contain high levels of NO, suggesting that the symbionts must overcome NO in order to colonize the light organ [56]. The detection of nitric oxide dioxygenase (Hmp), recently shown to play a role in NO detoxification, suggests that *V. fischeri* also maintains the ability to manage NO related stress in adult squid (Table 4) [53]. Once *V. fischeri* colonizes the light organ, nitric oxide synthase (NOS) is down-regulated and lower levels of NO likely allow the symbiont to grow in the crypt spaces under reduced RNS stress [56]. We identified NFκB repressing factor, which in addition to other immune functions, has been shown *in vitro* to negatively regulate transcription of NFκB pathway effectors, including NOS, by directly interacting with promoter region sequences (Table 5) [57], [58]. The results of this study provide a number of new host and symbiont targets involved in mediating ROS and RNS for further analyses.

The availability of iron has also been shown to be an important factor in squid/*Vibrio* symbioses [53], [59], [60]. Free iron plays a critical role in host/microbe interactions and under certain circumstances may allow development of pathogenic associations [61], [62]. Host proteins involved in sequestering free iron such as ferritin, transferrin, and melanotransferrin were identified (Table 5). These iron-binding proteins provide supporting evidence that iron remains limiting in the light organ and suggest a possible role for these proteins in regulating the growth of *V. fischeri* [59]. In contrast to the host, putative proteins that the symbiont may utilize for acquiring iron include receptors for the siderophores aerobactin and anguibactin (Table S3). Symbiont proteins involved in utilizing heme, another source of iron, are also present, and include HutZ, HutA, HuvX, and HmuT (Table S3). It is likely that *V. fischeri* employs several different strategies to meet its necessary iron requirements.

### Quorum Sensing

First described in *V. fischeri*, quorum sensing regulates bioluminescence, the light from which provides the host with an anti-predatory mechanism known as counterillumination [6], [63], [64]. Lux proteins involved in light production were identified and among the most abundant symbiont proteins (Table 3). Previous PAGE and transcriptomic analyses first revealed additional quorum sensing-regulated proteins, which were also detected by our characterization of the adult light organ proteome [26], [65]. QsrP is one of the most abundant proteins present in the symbiont proteome (Table 3), yet this novel protein remains functionally uncharacterized. Another quorum sensing-regulated protein identified in this study is a putative surface protein (VF_A0894) with immunoglobulin-like domains (Table 4). This putative surface protein is similar to the *Leptospira* immunoglobulin-like proteins (LigA, LigB and LigC) of pathogenic *Leptospira spp.*, which are thought to mediate adhesion to host cells [66]. These quorum sensing-regulated proteins may be important to a symbiotic lifestyle. We also detected LuxS, AI-2 synthase, which is involved in a second quorum sensing system in *V. fischeri* and has been implicated in regulating motility in *Vibrio alginolyticus* [67]–[69]. A link between LuxS and motility, may implicate a role for quorum sensing and the onset of motility prior to symbiont expulsion from the light organ (see below).

### Symbiont Signaling

Two-component signaling pathways are important mechanisms by which bacteria can sense the environment and have been identified in *V. fischeri* [70]–[73]. The roles in colonization for some of these regulators, which were present in our proteomic data (Table 4), such as GacA and ArcA, have been studied in detail, and mutagenesis of these genes has demonstrated that they are important in the association [71]–[73]. Although many regulators have already been characterized with respect to the symbiosis, many proteins involved in two-component signaling have unknown functions in the light organ. For example, CpxP, an abundant symbiont protein (Table 4), is a periplasmic component of *Escherichia coli* and *Vibrio cholerae* and involved in modulating the cell envelope stress response through CpxAR signaling, thus providing an appealing target for future studies [74], [75].

### Other Related Stresses

Although several were identified in this study, cold shock proteins have yet to be described with respect to the light organ symbiosis. Of the top 25 most abundant symbiont proteins present in the light organ, three were cold shock proteins (CspD, CspG, and VF_A0595; Table 3). Cold shock proteins often bind nucleic acids and function in general stress responses. Furthermore, they have been shown to play a role in regulating bacterial growth at stationary phase and may even serve as MAMPs recognized by hosts [76], [77]. One cold shock protein identified in the light organ, CspD, prevents replication from occurring in stationary phase *E. coli* cells by binding to single stranded DNA and blocking replication [78]. Prior to expulsion at dawn, the symbiont population is at its most dense during the day/night cycle. Therefore, cold shock proteins may play a role in either maintaining high cell densities in the light organ and/or assisting during the transition between the symbiotic and free-living state.

### Motility

Research involving the role of motility in the squid/*Vibrio* symbiosis has focused on the initiation of colonization. Within the light organ *V. fischeri* cells become differentiated with the loss of their flagella [79]. Upon release from the light organ at dawn, *V. fischeri* cells are believed to fully regenerate their flagella within several hours [79]. Our proteomic data show the presence and putative identification of several proteins related to flagellar structure including filamental proteins (FlaA, FlaC), basal body proteins (FlgB, FlgH, FlgI), and a motor protein (MotB; Table 4). Proteins related to flagellar regulation (FlrA and FlgM) and chemotaxis (CheW and CheZ) were also detected. A recent study indicated an increase in flagellar gene expression by light organ symbionts in the hours preceding dawn and *V. fischeri* mutants of FlaA and FlrA have been shown to be important for symbiotic competence [10], [80], [81]. FlrA was also found to be expressed by *V. fischeri* in the light organs of *E. scolopes* and a different squid species, *E. tasmanica*, but not in strains grown in seawater [82]. Together, the data from this present study and others suggests that *V. fischeri* cells are generating flagella prior to expulsion from the light organ and may be preparing for the transition from the symbiotic to the free-living state. Future studies should focus on signals in the changing microenvironment that may initiate this transition.

### Symbiont Metabolism

Within the light organ, *V. fischeri* employs a number of metabolic strategies [9], [10], [83], [84]. The daily rhythm of the light organ symbiont population coincides with fluctuations in symbiont metabolism [10]. Transcriptomics revealed a unique pattern in which during the night the symbiont ferments chitin as a means of obtaining energy. After the majority of the symbiont population is expelled from the light organ, the remaining symbionts anaerobically respire glycerol during the hours in which the light organ becomes replenished with a full symbiont population. The results of this study show abundant symbiont chitin binding proteins and chitinases, thus supporting these previous findings (Table S3). The diel shift in metabolism is one piece of evidence that supports the light organ as being a dynamic microenvironment that is under the regulation of both the host and symbiont [10].

### Summary

Proteomic studies of symbioses utilizing high-throughput techniques are becoming more common and have been used for analyses of the pea aphid-*Buchnera* symbiosis, nitrogen fixing symbioses of leguminous plants, human gut microbiota, and in characterizing the function of uncultivable symbionts in hydrothermal vent symbioses [85]–[89]. Characterization of the light organ proteome with high-throughput techniques allowed for the identification of a large number of host and symbiont proteins using little starting material and demonstrates the value of proteomic analyses in an effort to understand the relationship of a symbiotic association. The results of this study complement prior transcriptomic data, but have also identified a number of proteins of previously unknown function in the squid/*Vibrio* symbiosis [10]. The high-throughput techniques used here offer new methods for identification of host and symbiont proteins likely important for the maintenance of this and other host/microbe associations.

## Supporting Information

Figure S1 **Functional analysis of host and symbiont light organ proteomes.** **A.** COG category counts for all symbiont proteins present in the light organ (including putative identifications). **B.** KOG category counts for all host proteins present in the light organ (including putative identifications) using representative light organ ESTs. (COG/ KOG key: J- translation, ribosomal structure, and biogenesis, A- RNA processing and modification, K- transcription, L- replication, recombination and repair, B- chromatin structure and dynamics, D- cell cycle control, cell division and chromosome partitioning, Y- nuclear structure, V- defense mechanisms, T- signal transduction mechanisms, M- cell wall, membrane and envelope biogenesis, N- cell motility, Z- cytoskeleton, W- extracellular structures, U- intracellular trafficking, secretion and vesicular transport, O- posttranslational modification, protein turnover and chaperones, C- energy production and conversion, G- carbohydrate transport and metabolism, E- amino acid transport and metabolism, F- nucleotide transport and metabolism, H- coenzyme transport and metabolism, I- lipid transport and metabolism, P- inorganic ion transport and metabolism, Q- secondary metabolites biosynthesis, transport and catabolism, R- general function prediction only, S- function unknown).
(DOC)

Table S1 **Symbiont proteins detected in light organ exudates and central cores by MudPIT and LC MS/MS.**
(XLS)

Table S2 **BUDAPEST analysis of host proteins detected in light organ exudates and central core by MudPIT and LC MS/MS.**
(XLS)

Table S3 **Additional symbiont proteins detected in light organ exudates and central cores by MudPIT and LC MS/MS categorized by functions relevant to survival in the light organ crypts.**
(DOC)
