# Supplementary figures and images for: Characterizing the Host and Symbiont Proteomes in the Association between the Bobtail Squid, Euprymna scolopes, and the Bacterium, Vibrio fischeri
Source: PLoS One. 2011 Oct 5;6(10):e25649. doi: 10.1371/journal.pone.0025649 (PMC3187790; doi:10.1371/journal.pone.0025649)

**
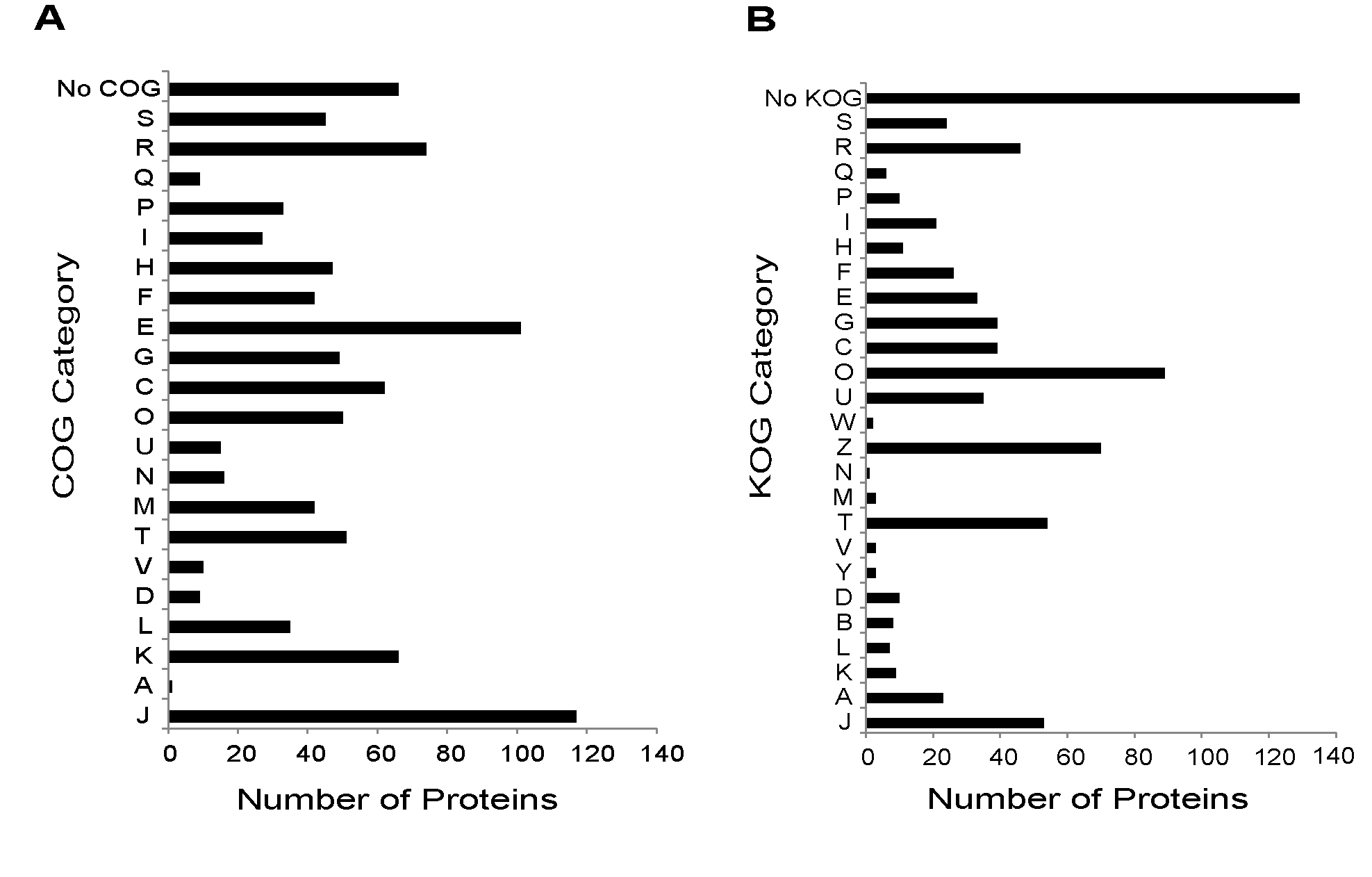
**

Supplement: Figure S1 — Functional analysis of host and symbiont light organ proteomes. A. COG category counts for all symbiont proteins present in the light organ (including putative identifications). B. KOG category counts for all host proteins present in the light organ (including putative identifications) using representative light organ ESTs. (COG/ KOG key: J- translation, ribosomal structure, and biogenesis, A- RNA processing and modification, K- transcription, L- replication, recombination and repair, B- chromatin structure and dynamics, D- cell cycle control, cell division and chromosome partitioning, Y- nuclear structure, V- defense mechanisms, T- signal transduction mechanisms, M- cell wall, membrane and envelope biogenesis, N- cell motility, Z- cytoskeleton, W- extracellular structures, U- intracellular trafficking, secretion and vesicular transport, O- posttranslational modification, protein turnover and chaperones, C- energy production and conversion, G- carbohydrate transport and metabolism, E- amino acid transport and metabolism, F- nucleotide transport and metabolism, H- coenzyme transport and metabolism, I- lipid transport and metabolism, P- inorganic ion transport and metabolism, Q- secondary metabolites biosynthesis, transport and catabolism, R- general function prediction only, S- function unknown). (DOC) [file pone.0025649.s001.doc]
