# Supplementary material for: Characterizing the Host and Symbiont Proteomes in the Association between the Bobtail Squid, Euprymna scolopes, and the Bacterium, Vibrio fischeri
Source: PLoS One. 2011 Oct 5;6(10):e25649. doi: 10.1371/journal.pone.0025649 (PMC3187790; doi:10.1371/journal.pone.0025649)
Supplement: Table S3 — Additional symbiont proteins detected in light organ exudates and central cores by MudPIT and LC MS/MS categorized by functions relevant to survival in the light organ crypts. (DOC) [file pone.0025649.s004.doc]

**Table S3:** Additional symbiont proteins detected in light organ exudates and central cores by MudPIT and LC MS/MS categorized by functions relevant to survival in the light organ crypts.

| **gi** | **ORF^a^** | **Gene** | **Functional Category and Protein Name** | **Score^b^** |
| --- | --- | --- | --- | --- |
|  |  |  | **Stress Response Related Proteins** |  |
| 59714277 | VF_A1094 | cspG | DNA-binding transcriptional regulator | 776 |
| 59712374 | VF_1767 | cspD | DNA replication inhibitor | 309 |
| 59712962 | VF_2355 | - | universal stress protein A | 283 |
| 59713778 | VF_A0595 | - | cold shock protein | 273 |
| 59711497 | VF_0890 | grxA | glutaredoxin 1 | 216 |
| 59712823 | VF_2216 | sspA | stringent starvation protein A | 163 |
| 59712907 | VF_2300 | - | glutaredoxin | 147 |
| 59714032 | VF_A0849 | yghU | putative glutathione S-transferase YghU | 130 |
| 59710690 | VF_0083 | uspA | universal stress global response regulator | 120 |
| 59711042 | VF_0435 | gshB | glutathione synthetase | 91 |
| 59713096 | VF_2489 | gor | glutathione reductase | 86 |
| 59711917 | VF_1310 | uspE | universal stress protein UspE | 79 |
| 59713168 | VF_2561 | - | cold shock protein | 67 |
| 59711390 | VF_0783 | ybbN | thioredoxin domain-containing protein | 38 |
| 59714004 | VF_A0821 | - | glutathione S-transferase | 26 |
| 59712103 | VF_1496 | - | glutaredoxin | 22 |
| 59714011 | VF_A0828 | - | glutaredoxin 2 | 22 |
|  |  |  | **Various Regulators** |  |
| 59712887 | VF_2280 | crp | cAMP-regulatory protein | 406 |
| 59711070 | VF_0463 | hlyU | transcriptional activator HlyU | 108 |
| 59712238 | VF_1631 | hns | DNA-binding transcriptional dual regulator H-NS | 106 |
| 59712875 | VF_2268 | metJ | transcriptional repressor protein MetJ | 104 |
| 59710817 | VF_0210 | - | transcriptional regulator | 93 |
| 59711498 | VF_0891 | yebK | DNA-binding transcriptional regulator HexR | 87 |
| 59711306 | VF_0699 | nrdR | transcriptional regulator NrdR | 80 |
| 59712760 | VF_2153 | glnK | nitrogen regulatory protein GlnK | 69 |
| 59711271 | VF_0664 | rpiR | DNA-binding transcriptional repressor | 66 |
| 59710913 | VF_0306 | zur | DNA-binding transcriptional repressor | 65 |
| 59712789 | VF_2182 | pdhR | transcriptional regulator PdhR | 51 |
| 59713619 | VF_A0436 | - | putative transcriptional regulatory protein | 49 |
| 59712185 | VF_1578 | ybaO | DNA-binding transcriptional regulator | 46 |
| 59711511 | VF_0904 | lrp | leucine-responsive transcriptional regulator | 30 |
| 59711688 | VF_1081 | - | TetR family transcriptional regulator | 25 |
| 59710936 | VF_0329 | ytfJ | transcriptional regulator | 24 |
| 59711145 | VF_0538 | csrA | carbon storage regulator | 22 |
| 59712152 | VF_1545 | yjiE | DNA-binding transcriptional regulator | 21 |
| 59712771 | VF_2164 | sfsA | sugar fermentation stimulation protein A | 21 |
| 59714331 | VF_A1148 | ycjZ | LysR family transcriptional regulator | 21 |
| 59713994 | VF_A0811 | malT | transcriptional regulator MalT | 19 |
| 172087698 | VF_1308 | fnr | transcriptional regulatory protein Fnr | 18 |
| 59712394 | VF_1787 | - | AraC family transcriptional regulator | 18 |
| 59713740 | VF_A0557 | sgrR | DNA-binding transcriptional regulator | 18 |
| 59712050 | VF_1443 | - | transcriptional regulatory protein | 17 |
| 59713441 | VF_A0258 | - | MerR family transcriptional regulator | 17 |
| 59713246 | VF_A0063 | yggD | putative DNA-binding transcriptional regulator | 15 |
|  |  |  | **ABC Transporters** |  |
| 59713125 | VF_2518 | dppA | dipeptide-binding protein DppA | 643 |
| 59713574 | VF_A0391 | - | ABC transporter amino acid-binding protein | 152 |
| 59712139 | VF_1532 | - | general L-amino acid-binding protein | 128 |
| 59711164 | VF_0557 | yjjK | putative ABC transporter ATP-binding protein | 108 |
| 59713280 | VF_A0097 | ynjB | putative ABC transporter solute-binding protein | 59 |
| 172087659 | VF_0397 | yrbC | organic solvent ABC transporter | 37 |
| 59711896 | VF_1289 | uup | ABC transporter ATPase component | 36 |
| 59713982 | VF_A0799 | malE | maltose ABC transporter periplasmic protein | 32 |
| 59712009 | VF_1402 | modA2 | molybdate ABC transporter | 29 |
| 59712201 | VF_1594 | oppD | oligopeptide transporter ATP-binding component | 25 |
| 59713126 | VF_2519 | dppD | dipeptide transport ATP-binding protein DppD | 21 |
| 59711299 | VF_0692 | - | oligopeptide transport ATP-binding protein OppF | 19 |
| 59713294 | VF_A0111 | ybhF | ABC transporter ATP-binding protein | 19 |
| 59713692 | VF_A0509 | - | multidrug resistance ABC transporter | 16 |
| 59713295 | VF_A0112 | ybhS | ABC transporter membrane protein | 15 |
| 59711491 | VF_0884 | - | ABC transporter ATP-binding protein | 14 |
| 59712410 | VF_1803 | - | magnesium transport ATPase protein C | 14 |
| 59713058 | VF_2451 | ftsX | ABC transporter membrane protein | 14 |
| 59713198 | VF_A0015 | - | ABC transporter ATP-binding protein | 13 |
|  |  |  | **Other Transporters** |  |
| 59712194 | VF_1587 | argT | lysine/arginine/ornithine transporter subunit | 246 |
| 59713268 | VF_A0085 | - | transporter | 225 |
| 59712054 | VF_1447 | rbsB | D-ribose transporter subunit RbsB | 211 |
| 59711941 | VF_1334 | - | oxalate/formate antiporter | 149 |
| 59713153 | VF_2546 | trkA | potassium transporter | 94 |
| 59710874 | VF_0267 | tbpA | thiamine transporter substrate binding subunit | 44 |
| 59712405 | VF_1798 | clcA | chloride channel protein | 29 |
| 59711884 | VF_1277 | - | transporter | 22 |
| 59712717 | VF_2110 | yaaJ | transporter | 21 |
| 59712420 | VF_1813 | - | long-chain fatty acid transport protein precursor | 17 |
| 59713905 | VF_A0722 | - | sodium/proton antiporter | 15 |
|  |  |  | **Outer Membrane Proteins** |  |
| 59711617 | VF_1010 | - | hypothetical protein VF_1010 | 362 |
| 59711082 | VF_0475 | ompU | Outer membrane protein U porin OmpU | 108 |
| 59712840 | VF_2233 | tolC | outer membrane channel protein | 86 |
| 59711570 | VF_0963 | pal | peptidoglycan-associated lipoprotein | 58 |
| 59712309 | VF_1702 | mltB | membrane-bound lytic murein transglycosylase B | 54 |
| 59714204 | VF_A1021 | sypB | outer membrane protein | 26 |
| 59711696 | VF_1089 | - | immunogenic protein | 24 |
| 59711816 | VF_1209 | - | membrane metalloprotease | 24 |
| 59711513 | VF_0906 | lolA | outer-membrane lipoprotein carrier protein | 20 |
| 172087680 | VF_0960 | tolA | membrane anchored protein | 18 |
| 59713242 | VF_A0059 | - | TonB-dependent outer membrane receptor | 16 |
| 59713650 | VF_A0467 | - | permease | 14 |
| 59713349 | VF_A0166 | qmcA | protease, membrane anchored | 13 |
|  |  |  | **Secretion Related Proteins** |  |
| 59712740 | VF_2133 | - | periplasmic component of efflux system | 101 |
| 59712800 | VF_2193 | secA | preprotein translocase subunit SecA | 80 |
| 59713060 | VF_2453 | ftsY | fused Signal recognition particle (SRP) receptor | 48 |
| 59711569 | VF_0962 | tolB | translocation protein TolB | 45 |
| 59711206 | VF_0599 | syd | SecY interacting protein Syd | 25 |
| 59711124 | VF_0517 | tadB1 | TadB-like protein | 20 |
| 59711123 | VF_0516 | tadA1 | putative type IV secretion NTPase | 19 |
| 59713082 | VF_2475 | gspC | general secretion pathway protein C | 14 |
|  |  |  | **Siderphore Related Proteins** |  |
| 59713348 | VF_A0165 | iutA | ferric aerobactin receptor precursor IutA | 300 |
| 59713347 | VF_A0164 | iucD | aerobactin siderophore biosynthesis protein IucD | 93 |
| 59713345 | VF_A0162 | iucB | aerobactin siderophore synthesis protein IucB | 60 |
| 59714010 | VF_A0827 | vctP | ferric anguibactin-binding protein | 51 |
|  |  |  | **Heme Metabolism** |  |
| 59711829 | VF_1222 | hmuT | hemin-binding periplasmic protein HmuT precursor | 111 |
| 59711834 | VF_1227 | huvX | HuvX protein | 109 |
| 59713009 | VF_2402 | hemE | uroporphyrinogen decarboxylase | 102 |
| 59711841 | VF_1234 | hutA | heme transport protein HutA | 78 |
| 59710660 | VF_0053 | hemB | delta-aminolevulinic acid dehydratase | 66 |
| 59710673 | VF_0066 | hemC | porphobilinogen deaminase | 54 |
| 59712138 | VF_1531 | - | ferrochelatase | 35 |
| 59711835 | VF_1228 | hutZ | HutZ protein | 33 |
| 59711401 | VF_0794 | hemH | ferrochelatase | 25 |
| 59713741 | VF_A0558 | - | coproporphyrinogen III oxidase | 23 |
| 59710687 | VF_0080 | hemN | coproporphyrinogen III oxidase | 22 |
|  |  |  | **Other Iron Transport and Binding Proteins** |  |
| 59712758 | VF_2151 | - | iron(III) ABC transporter | 1109 |
| 59710691 | VF_0084 | ftnA | ferritin iron storage protein (cytoplasmic) | 352 |
| 59713374 | VF_A0191 | - | FhuE receptor precursor | 89 |
| 59713093 | VF_2486 | cyaY | frataxin-like protein | 64 |
| 59711440 | VF_0833 | feoA | ferrous ion transport protein A | 33 |
|  |  |  | **Chemotaxis Related Proteins** |  |
| 59712433 | VF_1826 | cheW | chemotaxis coupling protein CheW | 140 |
| 59712439 | VF_1832 | cheZ | chemotaxis regulator CheZ | 33 |
| 59713352 | VF_A0169 | - | methyl-accepting chemotaxis protein | 26 |
| 59713860 | VF_A0677 | - | methyl-accepting chemotaxis protein | 24 |
| 59712385 | VF_1778 | - | methyl-accepting chemotaxis protein | 21 |
| 59711745 | VF_1138 | tcpI | methyl-accepting chemotaxis protein | 17 |
|  |  |  | **Chitin Metabolism** |  |
| 59712964 | VF_2357 | nagB | glucosamine-6-phosphate deaminase | 288 |
| 59712746 | VF_2139 | - | chitooligosaccharide-binding protein | 256 |
| 59711414 | VF_0807 | nagA | N-acetylglucosamine-6-phosphate deacetylase | 220 |
| 59712205 | VF_1598 | - | exochitinase | 216 |
| 59713196 | VF_A0013 | - | chitin-binding protein | 187 |
| 59711413 | VF_0806 | nagC | DNA-binding transcriptional dual regulator | 178 |
| 59713676 | VF_A0493 | nagZ | beta-hexosaminidase | 76 |
| 59713898 | VF_A0715 | - | chitodextrinase precursor | 65 |
| 59711593 | VF_0986 | - | chitodextrinase | 64 |
| 59712015 | VF_1408 | nagK | N-acetyl-D-glucosamine kinase | 27 |
| 59712754 | VF_2147 | - | N,N'-diacetylchitobiose phosphorylase | 27 |
| 59711666 | VF_1059 | - | basic endochitinase | 22 |
| 59712745 | VF_2138 | chiS | chitin sensor histidine kinase ChiS | 22 |
| 59712748 | VF_2141 | - | chitooligosaccharide permease protein | 18 |

a. Open reading frame (ORF) locations of the respective genes on the chromosomes of *V. fischeri*.

b. Scores were assigned by Mascot. Scores greater then 48 were significant while ones below 48 were considered putative identifications. Refer to Table S1 for more information.
